# Li_3_UHO_3_: A Uranium Hydride Oxide

**DOI:** 10.1021/acs.inorgchem.6c02161

**Published:** 2026-07-10

**Authors:** Marvin Michak, Kurt Weber, Paul Sicher, Marko Bertmer, Holger Kohlmann

**Affiliations:** † Faculty of Chemistry, Institute of Inorganic Chemistry and Crystallography, Leipzig University, Johannisallee 29, Leipzig 04103, Germany; ‡ Faculty of Physics and Earth System Sciences, Felix Bloch Institute of Solid State Physics, Leipzig University, Linnéstraße 3. Leipzig 04103, Germany

## Abstract

Li_3_UHO_3_, the first crystallographically characterized uranium hydride oxide (oxyhydride), was synthesized via a salt-flux reaction of UO_2_ with excess LiH at 973 K as a brown, air-sensitive solid. Single-crystal X-ray diffraction reveals a novel coloring variant of the rocksalt structure (*Pm* 3 *m*) in which oxide and hydride anions form a cubic close-packing and U^4+^/Li^+^ cations fill alternating octahedral voids. The edge-sharing [LiH_2_O_4_] and [UO_6_] octahedra yield ordered anion positions. Li–O distances are consistent with related hydride oxides, whereas Li–H separations are unusually long, reflecting weaker hydride coordination within edge-sharing octahedra. ^1^H/^7^Li MAS NMR and CHN analyses confirm H^–^ incorporation. Li_3_UHO_3_ thus extends heteroanionic hydride chemistry into the actinide series.

## Graphical abstract

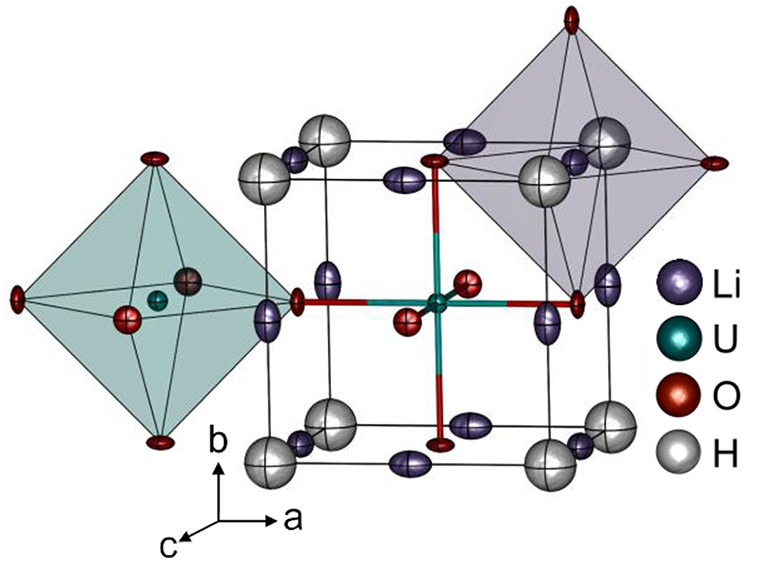

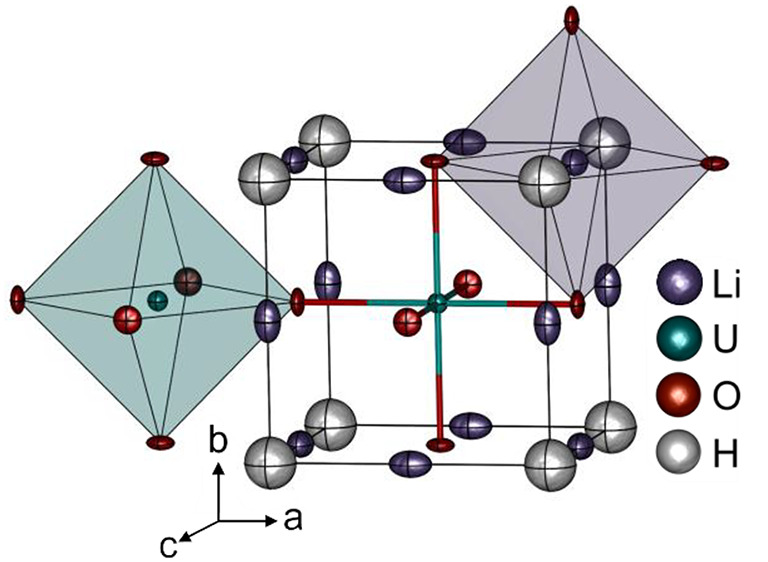

## Introduction

Heteroanionic compounds—ionic solids containing more than one type of anion—have attracted increasing interest as functional materials.^1^ Compared with cation substitution, using multiple anions is a comparatively underexplored strategy to tailor crystal structures and properties of solids, offering additional degrees of freedom to tune cation coordination environments (e.g., number, size, or relative spatial arrangement of neighboring anions).^1^ The hydride ion (H^–^) is particularly noteworthy: its large polarizability causes considerable variability in ionic radius and allows it to take part in chemical bonding of all types—from ionic over covalent to metallic.^2^ Introducing the oxide ion as a secondary anion establishes the substance class of hydride oxides (oxyhydrides), which have been discussed as materials for optical and data or energy storage applications,^3^ in catalysis,^4^ or as precursor materials to other heteroanionic materials.^5^ Well-established systems include for example ternary rare-earth hydride oxides *RE*HO (*RE* = Y,^6^ Dy,^7^ Er,^7^ Lu,^7^ Ho^8^), which have been studied in great detail. By contrast, reports on analogous actinide compounds are scarce. Although their immediate applications may be limited, actinide hydride oxides (oxyhydrides) are scientifically attractive because actinides can access multiple oxidation states and exhibit 5*f*-electron covalency, providing crystal-chemical degrees of freedom that may yield new structure types not available in the rare-earth series. Ternary actinide hydride oxides *An*HO (*An* = actinide metal) have primarily been invoked in discussions of actinide corrosion and nuclear-waste chemistry.^9^ Concerns have been raised about the possible pyrophoricity of hydridic corrosion products.^10^ PuHO was assumed to form as a black solid during hydrolysis of plutonium metal or plutonium hydride. The chemical composition was assessed on the basis of thermogravimetric analyses in the corrosion process,^9^ and it was assumed to further be oxidized to a compound denoted Pu_7_O_9_H_3_ of unknown structure.^9^ A uranium analogue reported as UO_1.80_H_0.30_ has also been proposed.^9^ These phases, however, remain poorly characterized, particularly crystallographically; PuHO has been described as related to the fluorite-type structure (*a* = 5.400(3) Å), a motif resembling some rare-earth hydride oxides (oxyhydrides), with similar comments for the uranium material.^9^

Other heteroanionic actinide hydrides are likewise uncommon. Thorium examples include hydride-containing bromide clusters (Th_6_Br_15_H_7_),^11,12^ nitride hydrides (ThNH,^13^ ThN_1.2_H^14^), and a poorly characterized hydrolysis product tentatively formulated as ThH­(*X*)O (X = OH, Cl).^15^ Additionally, several hydride carbides of thorium have been reported.^16^ Uranium studies are scarcer still: molecular UHF has been observed in a solid argon matrix,^17^ and gas-phase UH_2_O, UH_2_O_2_, and UHO­(OH) have been detected,^18^ but no solid-state analogues of these compositions have been established. Density-functional calculations further suggest that hydrogen in UO_2_ is energetically favored as H^–^ rather than as OH^–^, implying that uranium hydride oxides (oxyhydrides) are, in principle, accessible.^19^

Here, we report Li_3_UHO_3_, the first crystallographically well-characterized solid-state uranium hydride oxide (oxyhydride). Li_3_UHO_3_ forms as the major product when UO_2_ is reacted with excess LiH under salt-flux conditions. Single-crystal X-ray diffraction reveals a new structure type that can be described as a coloring variant of the rocksalt structure type.

## Experimental Details

Caution! Natural uranium was used in the experimental work described in this publication. Uranium contains exclusively radioactive isotopes, including long-lived alpha emitters (^235^U: *t*_1/2_ = 7.04·10^8^ a, ^238^U: *t*_1/2_ = 4.47·10^9^ a). Uranium compounds are additionally chemically toxic and must be handled with suitable care under approved institutional radiological safety procedures.

For synthesis and sample preparation, all samples were handled in an argon-filled glovebox, always maintaining the oxygen and water content below 0.5 ppm, if not stated otherwise. For the synthesis of UO_2_, which serves as a starting material for Li_3_UHO_3_, under atmospheric conditions, a sheet of uranium metal was cut into small pieces and dissolved in concentrated HNO_3_ (Carl Roth, 65%). The solution was boiled on a hot plate until the water had fully evaporated, after which yellow crystals of UO_2_(NO_3_)_2_·*n*H_2_O remained. The residue was subsequently dissolved in water and brought to a boil. Into this boiling solution, 35% H_2_O_2_ (OQEMA) was added, which resulted in the formation of UO_4_·H_2_O as a bright-yellow precipitate, which was subsequently filtered through a glass frit. This process was repeated until complete precipitation was reached. The precipitate in the glass frit was washed with water and acetone, transferred to a corundum crucible, and placed in a chamber furnace, where it was annealed at 350 °C for 12 h, yielding an orange powder. The obtained amorphous UO_3_ was subsequently reduced with H_2_ to UO_2_ by placing it on a silica glass boat, which was placed in a quartz glass tube within a tube furnace. The furnace was heated at 5 K/min to 700 °C with a dwelling time of 3 h. On the quartz glass tube, a steady gas stream of a mixture of 5% H_2_ and 95% Ar was applied. The product was subsequently cooled down under an atmosphere of this gas mixture. The reaction progress was monitored by XRPD.

A typical synthesis for Li_3_UO_3_H was conducted as follows: a stoichiometric excess of eight equivalents of LiH (abcr) was mixed with one equivalent of UO_2_ and thoroughly ground in an agate mortar until a homogeneous powder was achieved. The reaction mixture was subsequently pressed into a pellet of 5 mm diameter, which was then sealed in a V2A stainless steel ampule while being kept under inert conditions. This was achieved by welding a cap on both sides of a metal tube with a self-made welding machine at 10 A under an argon atmosphere of 400 mbar. Thereafter the ampule was sealed in a silica glass ampule and placed into a furnace. The furnace was heated at 100 K/h to either one of the tested temperatures, 973 and 1073 K; also, the annealing time was varied from 6 to 96 h. After the dwelling time, the furnace was cooled with the natural cooling rate. To monitor the reaction progress and to keep the mixture homogeneous, occasionally the ampule was opened, the reaction mixture was reground, and an XRPD measurement was conducted. Subsequently, the reaction was placed back into the furnace. This process was repeated for up to three times to reach a maximum turnover.

X-ray powder diffraction (XRPD) measurements were conducted using a Huber G670 diffractometer with Guinier geometry, a focusing Ge(111) monochromator, and Cu–K_α__1_ radiation. The image-plate detector was exposed for 15 min, and diffraction diagrams were summed over ten subsequent readout cycles.

XRPD data were analyzed using the software Topas, version 5 (Bruker AXS).^20,21^ The background was fitted by a 42nd-order Chebyshev polynomial. The zero error, scaling factor, lattice constants, microstructure, and atomic parameters (except for H atoms) as well as overall isotropic displacement parameters *B*_iso_ (assumed to be equal for each element in the sample and coupled to the main phase) were refined for phase ratios larger than 10 wt %. For smaller phase ratios, atomic parameters were taken from literature structures and *B*_iso_ were not refined and set to be equal to 1 Å^2^.

Single-crystal X-ray diffraction data were primarily collected using a Stoe STADIVARI diffractometer equipped with a microfocus X-ray tube emitting Ag–K_α1_ radiation (*λ* = 0.56086 Å) and a PILATUS 300 K pixel detector. During the measuring process, a flow of N_2_ gas streamed over the crystal to prevent contact with air. The resulting .xi-files were transcribed to .cbf-files using the silx-kit/FabIO module in Anaconda3 following the STOE Lab Note (Stoe and Cie GmbH: “Labnote—How to export XI images to CBF”).^22–24^ Integration and raw data processing including a decay correction were performed using CrysAlisPro 1.171.43.144a (Rigaku Oxford Diffraction, 2024),^25^ following the formula

$$K_{B}=e^{-2(B_{i}{\left(\frac{\sin\theta }{\lambda }\right)}^{2}+A_{i}(\frac{\sin\theta }{\lambda }))}$$

with *A*_*i*_ and *B*_*i*_ being the parameters refined for the decay correction. The empirical absorption correction was performed using the scaling algorithm SCALE3 ABSPACK implemented therein. Space group determination was carried out with Crysalis as well. Structure solution was performed with SHELXT, a dual-space method.^26^ For structure refinement via the least-squares method, the graphical user interface ShelXle^27^ for ShelXL^28^ was used.

Another refinement was performed using data reduced by the X-Area software without decay correction (Stoe and Cie GmbH, Darmstadt; Germany).^29^ The absorption correction was performed by the LANA program, and the space group determination was done with the X-Red32 program, both integrated into X-Area.^29^ Structure solution was performed with SHELXT, a dual-space method.^26^ For structure refinement via the least-squares method, the graphical user interface ShelXle^27^ for ShelXL^28^ was used. All structural visualizations were created using the Diamond program.^30^ Bond valence calculations were conducted in VESTA.^31^

The solid-state NMR spectra were recorded at room temperature using a Bruker Avance 400 (9.4 T) spectrometer. For this, a 4 mm MAS probe was used at a spinning frequency of 12 kHz. The ^1^H spectrum was acquired at 400.14 MHz with 128 scans, a 90° pulse length of 2.8 μs, and a repetition time of 1 s using the DEPTH sequence.^32^ ^7^Li MAS spectra were recorded at a frequency of 155.51 MHz with single-pulse acquisition and a pulse length of 3 μs with a recycle delay of 2 s. Referencing was done via the ^1^H channel according to the method described in ref 33. Deconvolution and plotting of the spectra were performed using DMFIT.^34^

## Results and Discussion

### Synthesis of Li_3_UHO_3_

The phase Li_3_UHO_3_ was formed during the reaction of a pelletized mixture of UO_2_ and excess LiH (1:8 molar ratio). The reaction mixture pellet was enclosed in a stainless-steel ampule, which was sealed by welding in an argon atmosphere. Heating the sample to 973 K and dwelling for different amounts of time with regular intermediate grinding and pressing into a new pellet yielded a dark-gray solidified product mixture containing single crystals of sufficient size for single-crystal X-ray diffraction measurements. Some of those crystals were isolated, and the rest of the sample was ground into a dark-brown powder. X-ray powder diffraction showed the formation of an unknown phase, which could be indexed with a cubic primitive cell, which did not correspond to any known phase containing the elements combined in the reaction mixture (*a* = 4.48291(2) Å, Figure 1).

**Figure 1:**
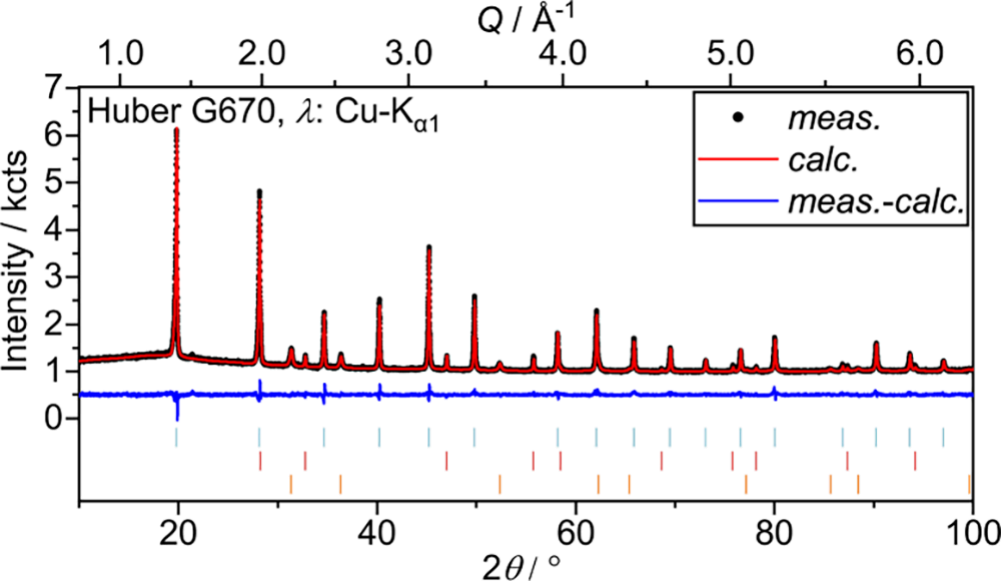
Rietveld refinement of the crystal structures of Li_3_UHO_3_, UO_2_, and UO based on X-ray powder diffraction data of the reaction product of UO_2_ with eight equivalents of LiH after 68 h at 973 K (Huber G670, λ: Cu–K_α1_ *R*_wp_ = 1.72%, *GoF* = 0.58). The Bragg markers denote, from top to bottom: Li_3_UHO_3_ (*Pm* 3 *m*, 83.1(1) wt %, *R*_Bragg_ = 1.66%, *a* = 4.48291(2) Å), UO_2_ (*Fm* 3 *m*, 6.70(7) wt %, *R*_Bragg_ = 0.843%, *a* = 5.46623(7) Å), and UO (*Fm* 3 *m*, 10.2(1) wt %, *R*_Bragg_ = 0.503%, *a* = 4.9407(2) Å). The difference plot was shifted by +0.5 kcts for better comparability.

Especially, the lattice parameter of the majority phase is significantly lower compared to UO_2_ (*a* = 5.47129(6) Å^35^). Additionally, broad reflections of another phase were observed in every reaction product attempted for the synthesis of the title compound. The reflections were satisfactorily fitted with the structure model of UO, which is reported to crystallize in the rocksalt structure type, with a similar lattice parameter as observed in our investigations, where it varied from sample to sample in the range of 4.923(1)–4.95411(5) Å (literature values: 4.92 and 5.00 Å).^36,37^ Another conceivable product, (Li_0.2_U_0.8_)­O_2_, crystallizes in the fluorite-type structure, showing a very different lattice parameter (*a* = 5.381 Å^35^) and can therefore also be excluded. Since the lattice parameter of the main phase, Li_3_UHO_3_, only varied in the range of 4.48291(2)–4.48907(3) Å over various prepared samples, the large variety in the lattice constant of the side product (UO) hints toward a phase width of this phase, which is why it will be formulated as UO_1+*x*_. However, due to the low crystallinity and phase share of this side phase, no more exact statements can be made regarding the chemical nature of this substance, for example, if secondary hydride anions are playing a role in this phase as well. The lattice parameter variation, i.e., the compositional variation of Li_3_UHO_3_, in contrast, seems to be very low if not negligible. Additionally, it was observed that leaving a prepared powder diffraction sample in air for several hours leads to a growing asymmetry and broadening of the reflections of the main phase, indicating that the substance is rather air sensitive and slowly hydrolyzes already at room temperature.

### Crystal Structure Analysis and Discussion

Brown, cuboid single crystals of Li_3_UHO_3_, sufficiently large to perform a single-crystal X-ray diffraction experiment, could be isolated from the reaction product. Due to decomposition of the crystal during the measurement, applying only a spherical absorption correction resulted in a high *R*_int_ value compared to *R*_σ_ (see Tables S1–S4). The resulting structure model showed high residual electron density maxima at the heavy atom positions at reasonable *R* values of the refinement (see Tables S1–S4). To account for the gradual decay of the crystal, a decay correction as implemented in CrysAlisPro was applied to the data set.^25^ This procedure resulted in a significant improvement of data consistency and consequently in much smaller residual electron densities and better bond precision and *R* values, which is why the results of the corrected data set are presented in the following (Tables 1–4). For comparison, the results of the structure refinement based on the nondecay-corrected data can be found in the Supporting Information.

**Table 1:** Crystal Structure Parameters of Li_3_UHO_3_ (*Pm* 3 *m*, *a* = 4.4913(1) Å) Based on Decay-Corrected Single-Crystal X-ray Diffraction Data (Figure 2) at 293(2) K

atom	site	*x*	*y*	*z*	*U*_iso_ */*Å	s.o.f.
U1	1*b*	$\frac{1}{2}$	$\frac{1}{2}$	$\frac{1}{2}$	0.00381(3)	1
O1	3*c*	0	$\frac{1}{2}$	$\frac{1}{2}$	0.0151(4)	1
Li1	3*d*	0	0	$\frac{1}{2}$	0.028(2)	1
H1	1*a*	0	0	0	0.07(5)	1

**Table 2:** Anisotropic Thermal Displacement Parameters (in Å^2^) of Li_3_UHO_3_

atom	*U*_11_	*U*_22_	*U*_33_	*U*_23_	*U*_13_	*U*_12_
O1	0.0053(6)	0.0199(6)	*U*_22_	0	0	0
Li1	0.018(2)	*U*_11_	0.047(5)	0	0	0

**Table 3:** Interatomic Distances in the Characteristic Polyhedra of the Metal Atoms in Li_3_UHO_3_

polyhedron	atoms	*d*/Å
[UO_6_] octahedron	U–O	2.24565(5)
[LiH_2_O_4_] octahedron	Li–O	2.24565(5)
	Li–H	2.24565(5)

**Table 4:** Crystallographic Data and Refinement Results for Li_3_UHO_3_

**sum formula**	**Li**_**3**_ **UHO**_**3**_
molar mass/g/mol	307.86
*T*/K	293(2)
space group	*Pm* 3 *m* (No. 221)
*a*/Å	4.4913(1)
*V*/Å^3^	90.597(6)
*Z*	1
X-ray density/g·cm^–3^	5.643
*μ*(Ag–K_α1_)/mm^–1^	38.700
diffractometer	STOE STADIVARI
radiation	Ag–K_α1_ (*λ* = 0.56083 Å)
2*θ*_min_; 2*θ*_max_/°	3.580; 31.243
*h*_min_, *h*_max_; *k*_min_, *k*_max_; *l*_min_, *l*_max_	–7, 8; −8, 8; −8, 5
total number of reflections	1774
unique reflections	90
*R*_int_; *R*_σ_	0.0369; 0.0141
data; restraints; parameters	90; 0; 7
goodness of fit (χ^2^)	1.186
*R*1(*F* ^2^ > 2σ(*F* ^2^))	0.0052
*wR*2 (all data)	0.0096
Δ*ρ*_max_; Δ*ρ*_min_/e·Å^–3^	0.493; −0.609
shape and color	brown cuboid

The crystal structure resulting from this experiment is depicted in Figure 2.^38^ It can be considered a coloring variant of the NaCl structure type, in which the anions O^2–^ and H^–^ form a cubic close-packed arrangement, while the cations U^4+^ and Li^+^ occupy the octahedral voids. The resulting [UO_6_] and [LiH_2_O_4_] octahedra are arranged in an alternating, edge-sharing manner, while octahedra of the same type are corner-sharing. The anion ratio of H^–^ to O^2–^ and the cation ratio of U^4+^ to Li^+^ is 1:3. In this way, the structure can be derived from a *klassengleiche* symmetry reduction of index 4 of the rocksalt-type structure (*Fm* 3 *m*), leading to the splitting of Wyckoff positions needed to fulfill the observed composition (*Pm* 3 *m*) (Figure 3). Alternatively, the structure can also be described as a filling variant of a partial *anti*-perovskite type. In that approach, Li^+^ ions occupy the octahedral voids that would remain empty in the ideal cubic perovskite structure, and the H^–^ ions occupy a position that would be occupied by a cation, making it a partial *anti*-type (Tables 1 and 2).

**Figure 2:**
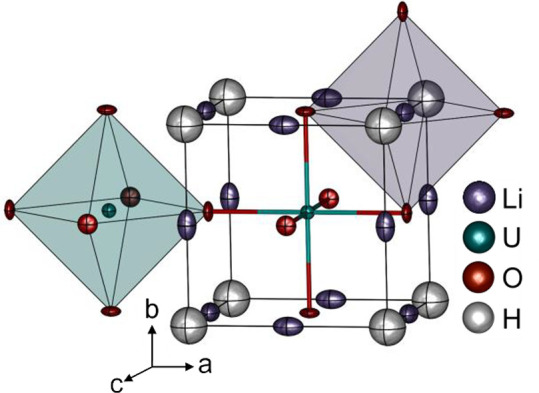
Crystal structure of Li_3_UHO_3_ within one unit cell. U and O, as well as Li and H atoms, each form a cubic-close packing arrangement, resulting in the overall structure being a coloring variant of the NaCl structure type. Thermal displacement ellipsoids are drawn at a 50% probability level.

**Figure 3:**
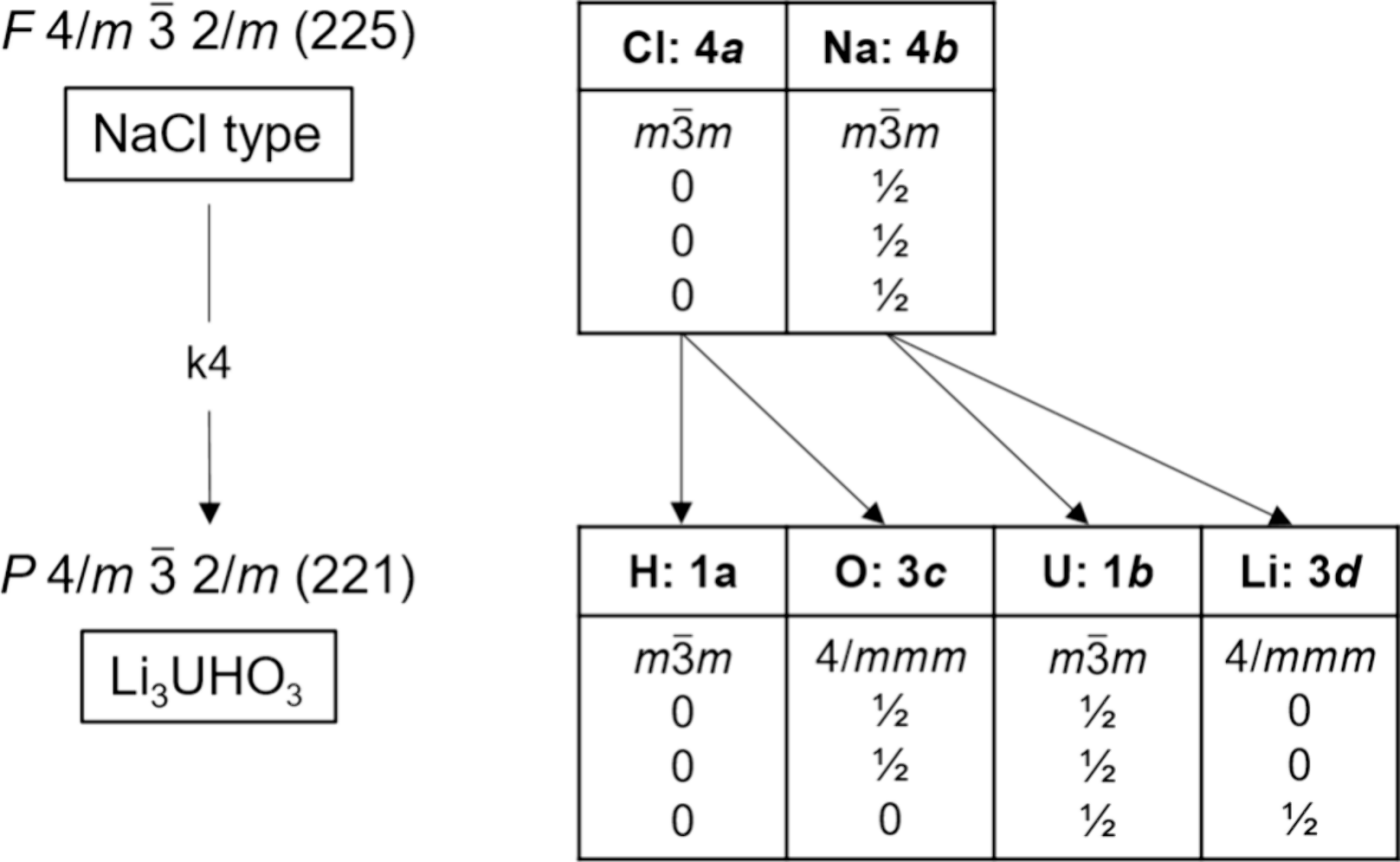
Bärnighausen tree demonstrating the crystallographic relationship of Li_3_UHO_3_ to the NaCl structure type.

The position of the H^–^-ion was assigned to a residual electron density in the difference Fourier analysis that corresponded to two electrons. Exchanging Li^+^ and H^–^, both of which have the same electron count, in the structure model leads to physically implausible *U*_iso_ values. Additionally, the structure model of Li_3_UHO_3_ presented herein features an alternating arrangement of cations and anions, as expected for the likely ionic and semiconducting brown solid, which would not be true for an inverse assignment of Li^+^ and H^–^. The oxygen atoms show a slightly prolate thermal vibration ellipsoid, with the vibration being oriented orthogonally to the U–O bond, which indicates a higher bond strength between O and U, than between O and Li. This could be due to a covalent bond share in this compound, typically seen in actinide chemistry due to the participation of the relativistically expanded 5*f* orbitals in chemical bonding. The shape of the ellipsoid is appropriate for a rocking vibration of the [UO_6_] octahedra. The lithium atoms appear to vibrate anisotropically as well, showing larger displacement along the Li–H axes, which is sensible for weaker bonding and the strong vibration of the small hydride ion in the rather large octahedral cavity, as shown by the large isotropic thermal vibration of the latter. Due to their high-symmetric Wyckoff positions, the hydrogen and uranium positions vibrate isotropically, which contributes to the overall low *R*1 value of the refinement, as the diffraction pattern is largely dominated by the scattering of the uranium atoms and its isotropic thermal displacement parameter is the only free parameter of the uranium atom in this structure model (Table 3).

Due to the occupation of special positions only, just one anion–cation distance of 2.24565(5) Å is observed within the two regular octahedra in Li_3_UHO_3_, as shown in Table 3. The U–O bond length falls within the expected range but lies toward the upper end relative to other [UO_6_] octahedra in lithium-containing compounds, as for example in Li_2_UO_4_ (*d*(U–O) = 2.1813 Å),^39^ LiUO_3_ (3× *d*(U–O) = 2.2807 Å, 3× *d*(U–O) = 2.0030 Å),^40^ Li_3_UO_4_ (2× *d*(U–O) = 2.245 Å, 4× *d*(U–O) = 2.125 Å),^41^ or Li_4_UO_5_ (2× *d*(U–O) = 2.2255 Å, 4× *d*(U–O) = 1.9916 Å).^42^ The mentioned compounds feature interatomic distances, which are marginally shorter than that observed in Li_3_UHO_3_. This could be attributed to the higher oxidation state in the respective compounds, when compared to Li_3_UHO_3_. Comparing the interatomic distance to a tetravalent, octahedrally coordinated uranium compound like BaUO_3_ (*d*(U–O) = 2.1945 Å),^43^ the discrepancy is smaller, but the comparability is lower, since the ionic radii of Ba^2+^ (*r*_eff_ = 1.61 Å for CN 12) and Li^+^ (*r*_eff_ = 0.76 Å for CN 6) differ significantly.^44^ The comparison to UO_2_, which possesses a much longer interatomic distance (*d*(U–O) = 2.3921 Å), is also not the most reliable point of comparison, since the coordination number in UO_2_ is eight instead of six. It can therefore be stated that the U–O distance is longer than in [UO_6_] octahedra of hexavalent uranium, which indicates a lower oxidation state in Li_3_UHO_3_. This is consistent with the result of the calculated bond-valence sum (BVS), which yields a value of 4.047.^45^ Nevertheless, the value is well within the expected range of distances between tetravalent uranium and oxide anions. Because uranium is only surrounded by oxide ions, the compound could also be named a uranate hydride, emphasizing the relationship toward similar compounds; however, the presence of the second anion makes Li_3_UHO_3_ unique within this class.

The Li–O distances in Li_3_UHO_3_ fall within the expected range for lithium-containing hydride oxides, including the quaternary phases LiSr_2_H_3_O (*d*(Li–O,H) = 2.2388 Å, *d*(Li–H) = 1.8614 Å),^46^ LiNd_2_HO_3_ (*d*(Li–O) = 2.2383 Å, *d*(Li–H) = 1.7427 Å),^47^ as well as LiLa_2_HO_3_ (*d*(Li–O) = 2.2965 Å, *d*(Li–H) = 1.7882 Å).^46^ However, the Li–H distances in Li_3_UHO_3_ are comparatively long. Direct comparison with LiSr_2_H_3_O is complicated by the 1:1 mixed oxygen/hydrogen atom occupancy at the position adjacent to lithium atoms, whereas Li_3_UHO_3_ exhibits ordered anion positions. In LiLa_2_HO_3_, which contains ordered but distorted [LiO_4_H_2_] octahedra, the Li–H distances are also significantly shorter than those observed in Li_3_UHO_3_. This difference is likely due to the distinct polyhedra connectivity patterns. In LiLa_2_HO_3_, the octahedra form layers by linking via common corners, resulting in linear coordination of H with Li atoms.^45^ In contrast, the edge-sharing octahedra in Li_3_UHO_3_ create an octahedral environment around hydride ions surrounded by lithium ions, leading to weaker bonding and longer interatomic distances. Notably, Li_3_UHO_3_ exceeds even the Li–H distances found in binary LiH (*d*(Li–H) = 2.042 Å),^48^ primarily due to the larger spatial requirements of the uranium atoms, which expand the unit cell and further elongate the Li–H distances. A comparably large distance is found in Li_3_AlH_6_ (*d*(Li–H) = 2.132 Å), which also features higher coordination numbers of H around Li and significant polyhedral distortion.^49^

So far, only a few quaternary compounds are known to share the Wyckoff sequence 221,*dcba*, raising the question of whether the structure of Li_3_UHO_3_ constitutes a new structure type. The (Ag_0.87_Sb_0.13_)­(Ag_0.15_Pb_0.44_Sb_0.41_)_3_Te_4_ type is the first example, which exhibits mixed cation occupancy on Wyckoff sites 1*b* and 3*d*.^50^ Moreover, a single anion species occupies the 1*a* and 3*c* sites, which is also true for ScPd_3_D_4_ (Table 5).^51^ The compound Li_0.5_La_0.5_TiO_3_ is characterized by strong underoccupancy of the lithium and lanthanum sites.^52^ Mo_0.75_N_0.5_ is another example with strong underoccupancy and like atoms on different Wyckoff sites (Table 5). Because of these pronounced differences, particularly in the anion distribution and overall structural chemistry, Li_3_UHO_3_ should not be assigned to any of the known structure types. It can, however, be described as a coloring variant of structure types like (Ag_0.87_Sb_0.13_)­(Ag_0.15_Pb_0.44_Sb_0.41_)_3_Te_4_ and ScPd_3_D_4_ or as a filling variant of the structure types of Mg_3_NF_3_, U_4_S_3_, and SrTiO_3_ (Table 5). Similarly, the structure can be viewed as two nested AuCu_3_-type arrangements (HO_3_/ULi_3_) that are shifted relative to each other by $\frac{1}{2}$ $\frac{1}{2}$ $\frac{1}{2}$ .

**Table 5:** Site Occupation in *Pm* 3 *m* (No. 221) Superstructures of the NaCl Type^a^

Wyckoff sequence	structure type	1*a*	3*c*	1*b*	3*d*
221,*dcba*	Li_3_UHO_3_	H	O	U	Li
221,*dcba*	*MM‘*_3_Te_4_	Te	Te	*M*	*M‘*
221,*dcba*	ScPd_3_D_4_	Sc	Pd	D	D
221,*dcba*	Li_0.5_La_0.5_TiO_3_	Ti	$\frac{1}{6}$Li	$\frac{1}{2}$ La	O
221,*dcba*	Mo_0.75_N_0.5_	Mo	$\frac{2}{3}$ Mo	N	$\frac{1}{3}$ N
221,*dca*	Mg_3_NF_3_,	N	F	□	Mg
221,*dca*	U_4_S_3_	U	U	□	S
221,*dba*	SrTiO_3_	Ti	□	Sr	O
221,*ba*	CsCl	Cl	□	Cs	□
221,c*a*	AuCu_3_	Au	Cu	□	□
221,d*a*	ReO_3_	Re	□	□	O
221,*dc*	NbO	□	Nb	□	O

a □ is the Schottky symbol for a vacancy (no occupation); *M* = (Ag_0.87_Sb_0.13_), and *M’* = (Ag_0.15_Pb_0.44_Sb_0.41_).

### NMR-Spectroscopic Investigation of Li_3_UHO_3_

To investigate whether hydrogen was contained in the new compound, ^1^H NMR spectra were recorded (Figure 4).

**Figure 4:**
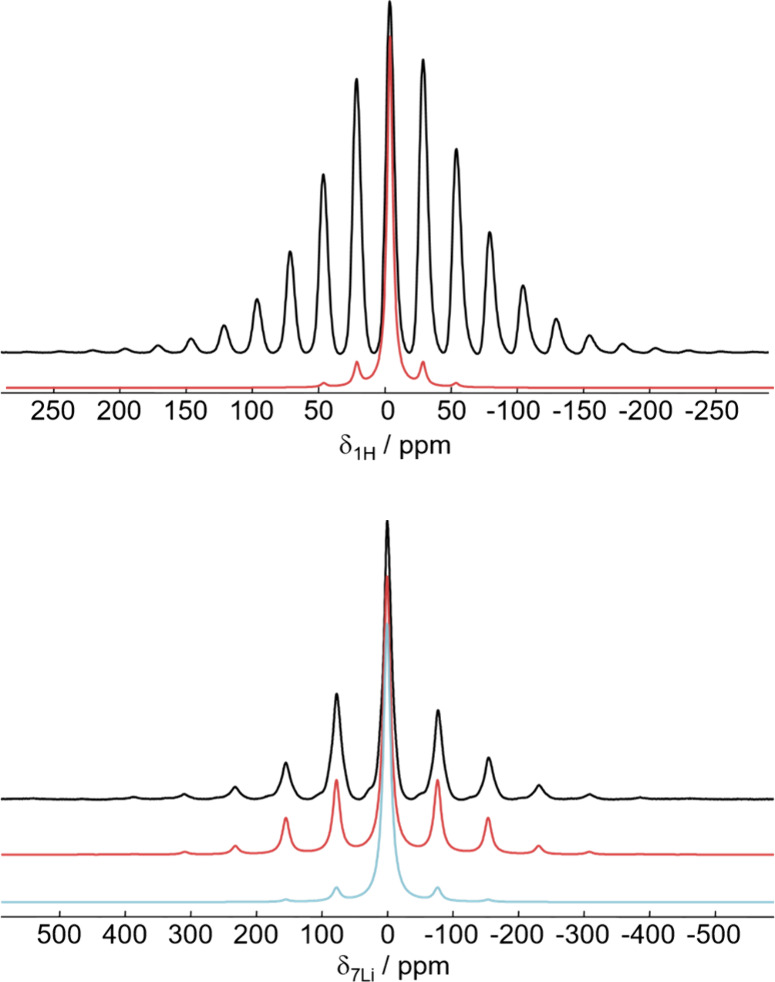
Top: black: ^1^H MAS spectrum; red: simulated spectrum with dipolar coupling to six nearest ^7^Li spins; bottom: ^7^Li MAS spectrum; red: simulated spectrum with dipolar coupling to two nearest ^1^H spins and quadrupolar coupling of 79 kHz; blue: simulated spectrum with dipolar coupling to two nearest ^1^H spins and quadrupolar coupling of 22 kHz spectra of the product mixture containing Li_3_UHO_3_.

A single signal was obtained with a chemical shift of −3.1 ppm and several spinning sidebands. The shift differs significantly from LiH at 2.9 ppm. Due to the line width of 4 kHz under MAS, small amounts of LiH signal hidden below the main line cannot be ruled out. The sideband manifold transfers into a static line width of about 50 kHz. Based on a second moment analysis^53,54^ from the crystal structure, a width of 34 kHz is expected from dipolar couplings to ^1^H and ^7^Li nuclei, which is in a similar range. The ^7^Li spectrum also shows a single line at 0 ppm with sidebands due to dipolar and quadrupolar couplings. LiH would resonate at 0.3 ppm. For the line width, both the second moment as well as the quadrupolar coupling was calculated according to the crystal structure. For the quadrupolar coupling, simple point charges were assumed for the surrounding atoms.^55^ A dipolar line width of 18 kHz and a quadrupolar coupling of 22 kHz were obtained (η = 0). Combining these results in a slightly lower sideband intensity than experimentally observed. However, for both ^1^H and ^7^Li, slight distortions of the hydrogen positions would easily increase the values for dipolar and quadrupolar couplings and could explain the discrepancies between calculations and the experiment. So, the NMR spectra go along with the X-ray analysis of the structure.

The combination of the observations and experimental data on Li_3_UHO_3_ (color, air sensitivity, crystal structure, and NMR spectra) provides clear hints of a semiconductor with U­(IV) and hydride anions, H^–^, according to a limiting ionic formula (Li^+^)_3_U^4+^H^–^(O^2–^)_3_.

## Conclusion

Li_3_UHO_3_ represents the first well-characterized uranium hydride oxide, establishing a new structure type within the mixed-anion materials landscape. Its crystal structure (space group *Pm* 3 *m*) features alternating edge-sharing [LiH_2_O_4_] and [UO_6_] octahedra with ordered hydride and oxide sites, yielding Li–O distances in line with known hydride oxides and rather long Li–H bond lengths forced by symmetry and the rather large uranium ions. NMR spectroscopic data confirm the presence of hydride ions, H^–^, in the crystal structure. Its chemical, optical, structural, and spectroscopic properties suggest Li_3_UHO_3_ to be a semiconductor with a limiting ionic formula (Li^+^)_3_U^4+^H^–^(O^2–^)_3_. While earlier investigations only provided vague indications of solid actinide hydride oxides, their existence has now been proven beyond doubt with Li_3_UHO_3_. Compared to the analogous rare earth hydride oxides, the early actinides have a much greater flexibility in terms of oxidation states. This gives hope for the development of a rich chemistry of actinide hydride oxides, maybe in the future also exhibiting direct interactions of actinides and hydride ions in heteroanionic systems. This unique combination would open avenues for investigating hydride ion behavior in 5*f*-electron systems.

## Supplementary Material



## Data Availability

All data analyzed for the present publication (integration and refinement results for the single-crystal X-ray diffraction measurements, raw data and refinement results for the X-ray powder diffraction data, raw and fit MAS NMR data, as well as all structural images and graphics presented in the publication) may be retrieved from the data repository RADAR4CHEM (doi:10.22000/dqdb5f1ke6paxkap).

## Notes

Results of the refinement of the structure of Li_3_UHO_3_ based on the nondecay corrected single-crystal X-ray diffraction dataset (PDF)
