# Supplementary material for: Li3UHO3: A Uranium Hydride Oxide
Source: Inorg Chem. 2026 Jul 10;65(29):16936–42. doi: 10.1021/acs.inorgchem.6c02161 (PMC13418178; doi:10.1021/acs.inorgchem.6c02161)
Supplement: Supplementary file 1 [file ic6c02161_si_001.pdf]

Supplementary Material

Li_3UO_3H – A Uranium Hydride Oxide

Marvin Michak*[a], Kurt Weber[a], Paul Sicher[a], Marko Bertmer[b] and Holger Kohlmann*[a]

[a] Marvin Michak, Kurt Weber, Paul Sicher, Prof. Dr. Holger Kohlmann
Faculty of Chemistry, Institute of Inorganic Chemistry and Crystallography
Leipzig University
Johannisallee 29, 04103 Leipzig
E-mail: marvin.michak@uni-leipzig.de, holger.kohlmann@uni-leipzig.de

[b] Dr. Marko Bertmer
Faculty of Physics and Earth System Sciences, Felix-Bloch Institute for Solid State Physics
Leipzig University
Linnéstraße 5, 04103 Leipzig, Germany

Results of the refinement of the structure of Li_3UHO_3 based on the non-decay corrected single-crystal X-ray diffraction dataset

Table S1: Crystallographic data and refinement results for Li_3UHO_3 refined on the basis of the non-decay-corrected dataset.

**sum formula**	$\mathbf{Li_3UHO_3}$
molar mass / g/mol	307.82
T / K	293(2)
space group	$Pm\overline{3}m$ (No. 221)
a / Å	4.4856(4)
V / Å^3	90.25(2)
Z	1
X-ray density / g·cm^{-3}	5.663
μ(Ag-K$_{\alpha1}$) / mm^{-1}	38.532
diffractometer	STOE STADIVARI
radiation	Ag-K$_{\alpha1}$ (λ = 0.56083 Å)
$2\theta_{min}$; $2\theta_{max}$ / °	5.072; 32.778
h_{min}, h_{max}; k_{min}, k_{max}; l_{min}, l_{max}	-8, 8; -8, 8; -5, 8
total number of reflections	1882
unique reflections	96
R_{int}; R_σ	0.0929; 0.0278
data; restraints; parameters	96; 0; 7
goodness of fit (χ^2)	1.126
$R1(F^2 > 2\sigma(F^2))$	0.0206
$wR2$ (all data)	0.0413
$\Delta\rho_{max}$; $\Delta\rho_{min}$ / e·Å^{-3}	2.210; -3.042
shape and color	brown cuboid

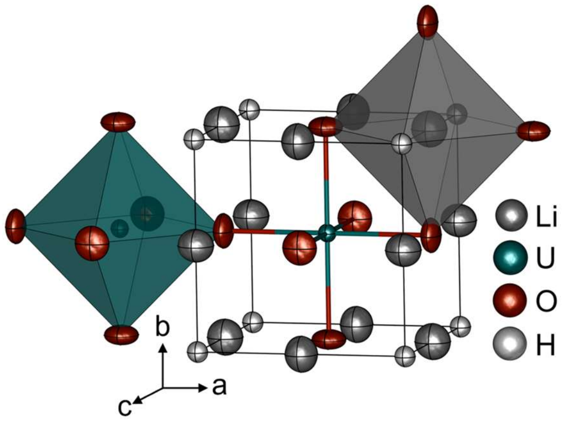

Figure S1. Crystal structure of Li_3UHO_3 within one unit cell as refined based on the non-decay-corrected dataset. U and O, as well as Li and H each form a cubic-close packing arrangement, resulting in the overall structure being a coloring-variant of the NaCl structure-type. Thermal displacement ellipsoids are drawn at a 50 % probability level.

Table S2. Crystal structure parameters of Li_3UHO_3 ($Pm\overline{3}m$, a = 4.4856(4) Å) based on the non-decay-corrected single-crystal X-ray diffraction data (**Figure S1**) at 293(2) K.

**Atom**	**Site**	***x***	***Y***	***z***	**U_{iso} / Å**	***s.o.f.***
U1	1*b*	½	½	½	0.0056(1)	1
O1	3*c*	0	½	½	0.016(1)	1
Li1	3*d*	0	0	½	0.022(5)	1
H1	1*a*	0	0	0	0.02(8)	1

Table S3. Interatomic distances in the characteristic polyhedra of the metal atoms in Li_3UHO_3 as obtained by refinement of the structure on the basis of non-decay-corrected single-crystal X-ray diffraction data.

polyhedron	atoms	d / Å
$[UO_6]$ octahedron	U-O	2.2428(2)
$[LiH_2O_4]$ octahedron	Li-O	2.2428(2)
	Li-H	2.2428(2)

Table S4. Anisotropic thermal displacement parameters (in $Å^2$) of Li_3UHO_3 as obtained by refinement of the structure on the basis of non-decay-corrected single-crystal X-ray diffraction data.

Atom	U_{11}	U_{22}	U_{33}	U_{23}	U_{13}	U_{12}
U1	0.0056(1)	0.0056(1)	0.0056(1)	0	0	0
O1	0.008(3)	0.022(3)	0.022(3)	0	0	0
Li1	0.023(7)	0.023(7)	0.02(1)	0	0	0
